# School Lunch Source and Adolescent Dietary Behavior

**Published:** 2009-09-15

**Authors:** Theresa A Hastert, Susan H. Babey

**Affiliations:** University of Washington Department of Epidemiology. At the time of this study, Ms Hastert was affiliated with the University of California at Los Angeles Center for Health Policy Research, Los Angeles, California.; University of California at Los Angeles Center for Health Policy Research, Los Angeles, California

## Abstract

**Introduction:**

As rates of childhood obesity rise, the nutritional content of lunches eaten at school is more heavily scrutinized. We examined the association between dietary behaviors and the number of days that adolescents bring lunch to school.

**Methods:**

We analyzed cross-sectional data for 2,774 adolescents who responded to the 2005 California Health Interview Survey and reported dietary behaviors for a weekday.

**Results:**

In bivariate analyses, adolescents who typically brought their lunch from home 5 days per week ate fast food on fewer occasions; consumed fewer servings of soda, fried potatoes, and high-sugar foods; and ate more fruit and vegetables compared with adolescents who never brought their lunch to school. In linear regressions controlling for demographics, body mass index, desire to change weight, parent education, and adult presence after school, students who typically brought their lunch to school 5 days per week ate fast food 0.35 fewer times and consumed 0.35 fewer servings of soda, 0.10 fewer servings of fried potatoes, 0.25 fewer servings of high-sugar foods, and 0.95 more servings of fruit and vegetables per day compared with students who never brought their lunch to school.

**Conclusion:**

These findings suggest that adolescents who bring lunch to school from home have more positive dietary behaviors than do adolescents who get their lunches from other sources. Improving the nutritional quality of foods offered from other sources, such as the National School Lunch Program and competitive foods, could help improve adolescent dietary behaviors.

## Introduction

Rising rates of childhood obesity have meant greater scrutiny of food environment and the role of foods eaten at school in overall dietary intake of children and adolescents. Many studies have examined the relationship between meals eaten as part of the National School Lunch Program (NSLP) and dietary behaviors and have found that NSLP participants consumed more fruit and vegetables; more fat, saturated fat, and sodium; and less sugar and soda than did nonparticipants (1,2). The NSLP establishes nutritional guidelines for all meals served to students under its auspices. For years, many foods sold outside of the NSLP — or "competitive foods" — have not been subject to similar guidelines. The availability of competitive foods in schools has been associated with negative dietary behaviors, including higher saturated fat intake (3), higher sweetened beverage intake (4), and lower fruit and vegetable intake (2-5). Because of concerns about the contribution of competitive foods to the overall diets of school-aged children, the Institute of Medicine recently released recommendations about appropriate nutrition standards and guidelines concerning the availability and consumption of competitive foods and beverages at school (6). States and school districts have begun to regulate the nutrition content of competitive foods (7).

Few studies have examined the nutritional makeup of lunches that students bring from home (8,9) or whether bringing lunch from home is associated with better or worse dietary behaviors compared with getting lunch from other sources. Some research suggests that lunches from home are lower in saturated fat and total fat than lunches sold in the middle school cafeteria (9). We are aware of only 1 study that compared the dietary behavior of students who bring lunch from home to those with other meal sources. This research found that students who ate a school lunch ate more fruit and vegetables than did those who brought lunch from home (10), but it focused on second- and fifth-grade students, who likely have less autonomy in their lunch decisions than the adolescents we studied.

We examined several dietary behaviors, specifically number of times fast food was eaten and servings of soda, fried potatoes, desserts, fruit, and vegetables, of adolescents (aged 12-17 years) who bring their lunch to school frequently compared with those who rarely or never bring lunch to school. We tested the hypothesis that students who regularly bring lunch to school from home consume less soda, fast food, fried potatoes, and desserts and more fruit and vegetables.

## Methods

### Data source and participants

This study uses cross-sectional data from the adolescent portion of the 2005 California Health Interview Survey (CHIS) (N = 2,774). The CHIS is a statewide random-digit–dialed telephone survey of California's noninstitutionalized population living in households. One randomly selected adult (aged 18 years or older) was interviewed in each household. In households with adolescents (aged 12-17 years), 1 adolescent was randomly selected to be interviewed directly. Adolescent interviews were conducted after obtaining permission from the adolescent's parent or guardian and consent from the adolescent. Interviews were conducted in English, Spanish, Chinese (Mandarin and Cantonese dialects), Vietnamese, and Korean. Detailed information about CHIS methods is available elsewhere (11). The Office for the Protection of Research Subjects at the University of California at Los Angeles approved this research.

### Outcome variables

Mean dietary intake was calculated from the following questions: "Yesterday, how many servings of fruit, such as an apple or a banana, did you eat?"; "Yesterday, how many servings of vegetables, like corn, green beans, green salad or other vegetables did you eat?"; "Yesterday, how many servings of French fries, home fries, or hash browns did you eat?"; "Yesterday, how many glasses or cans of soda, such as Coke, or other sweetened drinks, such as fruit punch or Sunny Delight did you drink? Do not count diet drinks."; "Yesterday, how many glasses of 100% fruit juice such as orange juice or apple juice did you drink?"; "Yesterday, how many servings of high-sugar foods, such as cookies, candy, doughnuts, pastries, cake or popsicles did you have?"; and "Yesterday, how many times did you eat fast food? Include fast food meals eaten at school, at home, or at fast-food restaurants, carryout, or drive-through." Servings of fruit were combined with servings of fruit juice to assess overall fruit consumption. In addition, servings of vegetables, fruit, and fruit juice were combined to assess overall fruit and vegetable consumption.

### Correlates

Adolescents were asked, "During the school year, about how many times a week do you usually bring your own lunch to school from home?" In addition to the information about bringing lunch from home, we assessed adolescent and parent characteristics as correlates. Demographic information included age, sex, race/ethnicity, household income, the educational attainment of the adult respondent from the adolescent's household, how frequently an adult was present after school, whether 1 or both of the adolescent's parents were born outside of the United States, adolescent weight, and whether the adolescent respondent was currently trying to change his or her weight. Age, sex, and race/ethnicity were self-reported by the adolescent. Adolescents also self-reported whether they were trying to lose or gain weight, maintain weight, or not do anything to change weight and how frequently an adult was present after school.

Adolescent body mass index (BMI) was calculated on the basis of the adolescent's self-reported height and weight. Age- and sex-specific growth charts from the Centers for Disease Control and Prevention were used to classify adolescents as overweight (BMI ≥95th percentile for their age and sex), at risk for overweight (BMI ≥85th percentile but <95th percentile), normal weight (BMI ≥5th percentile but <85th percentile), or underweight (BMI <5th percentile) (12).

The adolescent's parent or guardian reported the country of birth of both of the adolescent's parents and total household income and the number of residents in the household, which were used to calculate income as a percentage of the federal poverty level (FPL). In 2005, the FPL was $12,755 annually for a family of 2 and $19,971 annually for a family of 4 (13). These thresholds were used to categorize household income as below 185% FPL, 185% to 299% FPL, and 300% FPL or above. Adolescents from households with incomes below 185% FPL are eligible for free or reduced-price lunch through the NSLP (14).

### Statistical analyses

Data were analyzed with SAS version 9.1 (SAS Institute, Inc, Cary, North Carolina) and SUDAAN version 9.0.3 (RTI International, Research Triangle Park, North Carolina) to account for the complex survey design of the CHIS. Weighted bivariate analyses and 2-tailed *t* tests were conducted to examine the unadjusted association between number of days bringing lunch and dietary behaviors. Weighted linear regression models were used to determine whether associations could be made after adjusting for the demographic and other characteristics of the adolescents and parents. Analyses were conducted in 2007 and 2008. Significance was set at *P* < .05.

## Results

### Sample characteristics

The 2005 CHIS collected data from 43,020 adults and 4,029 adolescents from July 2005 through April 2006. The completed interview rate for adolescents was 48% among screened households. The sample was limited to adolescents who provided dietary intake information for a school day (N = 2,774) (Table 1).

### Dietary behaviors

Adolescents reported that on the previous day they had consumed fast food an average of 0.56 times and had consumed, on average, 1.16 servings of soda, 0.28 servings of fried potatoes, 1.29 servings of high-sugar foods, and 4.05 servings of fruit and vegetables (Table 2). In bivariate analyses, adolescents who typically brought their lunch to school from home 5 days per week consumed fast food fewer times and consumed fewer servings of soda, fried potatoes and high-sugar foods, and more fruit and vegetables compared with adolescents who never brought their lunch to school. Students who typically brought their lunch to school between 1 and 4 days per week also consumed fast food significantly fewer times and consumed fewer daily servings of soda and more daily servings of fruit and vegetables compared with students who never brought their lunch to school. In linear regression analyses, for every additional day per week that adolescents reported bringing their lunch to school from home, they reported eating fast food 0.07 fewer times and consuming 0.07 fewer servings of soda, 0.02 fewer servings of fried potatoes, 0.05 fewer servings of high-sugar foods, and 0.19 more servings of fruit and vegetables (Table 3). Assuming that consumption on the previous day was representative of typical behavior, students who reported that they typically bring their lunch to school 5 days per week eat fast food 0.35 fewer times per day and consume 0.35 fewer servings of soda, 0.10 fewer servings of fried potatoes, 0.25 fewer servings of high-sugar foods, and 0.95 more servings of fruit and vegetables per day compared with students who typically do not bring their lunch to school. We adjusted for attempting to change weight and for weight status; however, days bringing lunch from home remained associated with dietary behavior.

## Discussion

These findings suggest that bringing lunch to school from home more often is associated with positive dietary behaviors in adolescents. In an average school week, a student who does not bring his or her lunch to school from home eats fast food 1.75 more times and consumes 1.75 more sodas, 0.50 more servings of fried potatoes, 1.25 more servings of high-sugar foods, and 4.75 fewer servings of fruit and vegetables compared with a student who brings his or her lunch from home every day. Although any of these behaviors might have a small effect on overall health or body weight on its own, taken together, these behaviors could put adolescents who typically do not bring lunch from home at increased risk of obesity and other negative health consequences compared with those who typically bring lunch from home every day.

This study compares the dietary behaviors of adolescents who frequently bring lunch from home with those who get lunch from other sources. Previous research has examined the nutritional content of NSLP meals (15,16) and competitive foods (17) served in schools, as well as the association between NSLP participation and mostly positive dietary behaviors compared with nonparticipants (1,2) and the association of competitive food availability and consumption with mostly negative dietary behaviors (4,5,18-20). We are aware of little information comparing the dietary behaviors of students who bring lunch from home to those of students who get their lunches from other sources (9).

Our finding that adolescents who typically bring lunch from home consume more fruit and vegetables does not agree with previous research that found that students who typically ate school lunch consumed more fruit and vegetables than those who brought lunch from home (10). This discrepancy could be due to an older study population (aged 12-17 years in the current study compared to aged 6-12 years in the previous research), who are likely to have more lunch options outside the NSLP. Additionally, where the previous research compared dietary behaviors of children who ate school lunch with those who brought lunch from home, we did not know whether the adolescents who did not bring lunch from home ate the NSLP lunch or got their lunches from other sources, such as competitive foods or a fast-food restaurant; most students likely get lunch through some combination of the NSLP and competitive food offerings at school. Nationally, 94% of students aged 5 to 18 years attend schools that serve NSLP meals. Among these, 58% eat them 5 days each week and an additional 21% participate at least once per week (21). Almost all middle and high schools (98% and 94%, respectively) offer competitive foods during breakfast or lunch; weekly competitive food revenues are $1,760 per 1,000 students in middle schools and $1,985 per 1,000 students in high schools (16).

Bringing lunch from home could reflect greater health consciousness on the part of adolescents or their parents. Students who are the most health-conscious likely have the most positive overall dietary behaviors, and these students may bring lunch to school more often than other students, which would strengthen the observed relationship between positive dietary behaviors and the number of days adolescents bring lunch from home. Previous research has shown that weight-control behaviors in adolescents are associated with different mean intakes of a number of foods and nutrients (22) and that weight status is related to dietary behaviors (23).

This study has several limitations. The dietary intake data are self-reported, making them subject to errors. A single question was used to address each dietary behavior, and questions ask about diet on the previous day, which might not be representative of the respondents' overall diet patterns. However, in this sample these measures should provide meaningful differentiation in terms of dietary behavior. In addition, questions about dietary behavior based on a single day are expected to represent typical daily dietary behavior when taken in the aggregate. Additionally, we did not assess or control for the social desirability of bringing lunch. 

Our results suggest that the lunches students get at school, either through the NSLP or competitive foods, are associated with worse overall dietary behaviors compared with lunches students bring from home. This points to a potential opportunity to improve adolescent dietary behaviors by improving the nutritional content of foods served at schools. These improvements could come in the form of restricting unhealthy options, providing healthier alternatives, or a combination of the two. Since these data were collected, California enacted legislation regulating the nutritional content of all competitive foods and beverages sold in schools. Future research examining the relationship between bringing lunch to school from home and dietary behaviors after those regulations took effect could suggest whether that legislation had the desired effect on the dietary behaviors of California adolescents and could influence similar efforts in other locations.

Future research incorporating the meal sources of students who do not bring lunch from home would be valuable in identifying differences in dietary behaviors of adolescents who bring lunch to school compared with those who eat meals provided as part of the NSLP and who eat competitive foods separately. Further research examining the relationship between days bringing lunch from home and dietary behaviors stratified by sex could determine whether this relationship is different for boys and girls. Research into the association between lunch source and BMI would also be valuable.

These findings suggest that lunches from home are associated with positive dietary behaviors in adolescents compared with lunches from other sources. Because students who do not bring lunch from home are likely to frequently get lunch through some combination of competitive foods and the NSLP, improving the nutritional quality of competitive foods and foods offered through the NSLP could improve adolescent dietary behaviors.

## Figures and Tables

**Table 1:** Characteristics of California Adolescents Aged 12-17 Years, California Health Interview Survey, 2005

**Characteristic**	No. (%) (N = 2,774)^a^
Race/ethnicity	
White	1,458 (40)
Latino	765 (36)
Asian	234 (11)
African American	140 (7)
American Indian/Alaska Native	41 (2)
Other single race/multiple races	136 (4)
Age, y	
12	483 (17)
13	477 (16)
14	512 (19)
15	479 (18)
16	441 (16)
17	382 (14)
Sex	
Female	1,357 (49)
Male	1,417 (51)
Household income as percentage of federal poverty level^b^	
<185	813 (39)
185-299	433 (17)
≥300	1,528 (44)
Days bringing lunch to school in a typical week^c^	
0	1,479 (61)
1-4	722 (24)
5	539 (15)

a Numbers are unweighted, percentages are weighted. Percentages may not total 100% because of rounding. N is the sample of adolescents who provided dietary information for a school day.

b In 2005, the federal poverty level was $12,755 annually for a family of 2 and $19,971 for a family of 4 (13).

c Only asked of adolescents attending school.

**Table 2:** Dietary Intake as a Function of Days Bringing Lunch to School Among California Adolescents Aged 12-17 Years, California Health Interview Survey, 2005

Food or Beverage	All Adolescents Mean Servings/day^a^	Days Bringing Lunch to School in a Typical Week				

		0	1-4		5	

		Mean Servings/Day^a^	Mean Servings/Day^a^	*P* Value^b^	Mean Servings/Day^a^	*P* Value^b^
Fast food^c^	0.56	0.66	0.53	.01	0.29	<.001
Soda	1.16	1.32	1.06	.006	0.69	<.001
Fried potatoes	0.28	0.30	0.29	.89	0.15	<.001
High-sugar foods	1.29	1.32	1.29	.70	1.10	.02
Fruit^d^ and vegetables	4.05	3.79	4.35	.002	4.64	<.001

a Servings were reported as being consumed on the day before the survey.

b *P* values indicate differences from 0 days bringing lunch to school in a typical week and were calculated by using 2-tailed *t* tests.

c For this table, servings of fast food are equivalent to "times" fast food was consumed in the previous day.

d Includes fruit juice.

**Table 3:** Multivariate Linear Regression Analyses of Dietary Behaviors Among California Adolescents Aged 12-17 Years, California Health Interview Survey, 2005^a^

Characteristic	Servings of Fast Food	Servings of Fried Potatoes	Servings of Soda	Servings of High-Sugar Foods	Servings of Fruit^b^ and Vegetables
Age^c^	0.02	0.02^d^	0.07^e^	−0.03	−0.11^d^
Sex					
Female	−0.04	−0.06	−0.36^e^	0.01	−0.42^e^
Male	Reference				
Race/ethnicity					
Latino	0.15^d^	0.12^d^	0.23	0.10	0.06
Asian	0.27^e^	0.14	−0.28	0.07	0.58
African American	0.11	0.08	0.84^e^	0.16	0.40
American Indian/Alaska Native	−0.02	0.04	0.35	−0.30	−0.24
Other single race/multiple races	0.30	−0.04	0.11	0.32	−0.21
White	Reference				
Household income as percentage of federal poverty level^f^					
<185	0.01	0.00	0.17	0.07	0.00
185-299	0.09	0.03	0.24^d^	0.03	−0.21
≥300	Reference				
Responding parent's educational attainment					
Less than high school	0.22^d^	0.10	0.11	0.04	−0.36
High school graduate	0.09	0.15^e^	0.46^e^	−0.13	−0.20
Some college	0.07	0.11^d^	0.22^d^	−0.01	−0.47^d^
College graduate	Reference				
Adult present after school					
Almost never or never	−0.08	−0.07	0.01	−0.18	−0.76^e^
Some of the time	0.02	0.08	0.11	0.21	−0.33
Always or most of the time	Reference				
Parents' nativity					
Both parents born outside United States	−0.12	−0.13^d^	−0.08	−0.33^d^	0.39
One parent born outside United States	−0.08	−0.04	−0.09	−0.12	0.25
Both parents born in United States	Reference				
Weight status^g^					
Underweight	−0.11	−0.09	−0.15	0.21	0.10
Normal weight	Reference				
At risk for overweight	0.02	−0.04	−0.08	0.13	0.06
Overweight	−0.09	−0.04	−0.14	−0.28^d^	0.23
Currently trying to change weight					
Lose weight	−0.04	0.10^d^	−0.19	−0.25^e^	0.44^d^
Maintain weight	0.02	0.03	−0.07	0.02	0.47^d^
Gain weight	0.08	0.08	0.05	0.11	−0.15
Not doing anything to change weight	Reference				
Days bringing lunch to school from home^h^	−0.07^e^	−0.02^d^	−0.07^e^	**−**0.05^e^	0.19^e^

Abbreviation: BMI, body mass index.

a Servings were reported as being consumed on the day before the survey. For this table, servings of fast food are equivalent to "times" fast food was consumed in the previous day.

b Includes fruit juice.

c Difference in servings for each additional year of age over 12.

d *P* < .05.

e *P* < .01.

f In 2005, the federal poverty level was $19,971 for a family of 4 (13).

g Centers from Disease Control and Prevention growth charts were used to classify adolescents as overweight (BMI ≥95th percentile for their age and sex), at risk of overweight (BMI ≥85th percentile but <95th percentile), normal weight (BMI ≥5th percentile but <85th percentile), or underweight (BMI <5th percentile).

h Difference in servings for each additional day adolescents reported bringing lunch to school from home over 0 days per week.
